# Capacitive Phase Shift Detection for Measuring Water Holdup in Horizontal Oil–Water Two-Phase Flow

**DOI:** 10.3390/s18072234

**Published:** 2018-07-11

**Authors:** Hongxin Zhang, Lusheng Zhai, Cong Yan, Hongmei Wang, Ningde Jin

**Affiliations:** School of Electrical and Information Engineering, Tianjin University, Tianjin 300072, China; zhxlixue@tju.edu.cn (H.Z.); tjyancong@tju.edu.cn (C.Y.); whm712@tju.edu.cn (H.W.); ndjin@tju.edu.cn (N.J.)

**Keywords:** oil–water two-phase flow, capacitance sensor, phase shift detection, water holdup

## Abstract

In this paper, a phase shift detection system of flow impedance is designed based on a concave capacitance sensor (CCS). The flow impedance of oil–water stratified flow is investigated by establishing an equivalent circuit model and a finite element model. The influence of exciting frequency and sensor geometric parameters on the phase shift output of the CCS is studied to access an optimal phase shift measurement system. An experiment of horizontal oil–water two-phase flows was conducted during which four flow patterns are observed, i.e., stratified flow (ST), stratified wavy flow (SW), dual continuous flow (DC), and dispersed oil-in-water and water flow (DO/W&W). The phase shift responses of the CCS to the water holdup variation are collected. The results indicate that the phase shift response of the CCS presents satisfied sensitivity for ST and SW flow patterns, which is consistent with the predictions of the equivalent circuit model and the finite element model. Although the flow structures of DC and DO/W&W flows are extremely nonuniform, the phase shift response of the CCS still shows better linearity and sensitivity to the water holdup variation. In general, the capacitive phase shift detection technology exhibits advantages for water holdup measurement in horizontal oil–water two-phase flow with nonuniform phase distributions and conductive water.

## 1. Introduction

Horizontal oil–water two-phase flow widely exists in many important industrial production processes, such as those relating to petroleum, chemical engineering, and nuclear systems. Due to the effect of gravity, oil–water flow presents stratified nonuniform flow structures in pipelines. For low oil and water flow velocity, stratified (ST) flow and stratified wavy (SW) flow can be observed in the horizontal pipe. With the oil–water flow velocity increasing, dispersed oil and water drops are entrained in the continued water and oil phase, respectively. This flow pattern is defined as dual continuous (DC) flow. When the water superficial velocity is much higher than that of the oil phase, the oil phase is broken into a dispersed form and the water phase is continuous. This flow pattern is dispersed oil-in-water and water (DO/W&W) flow.

As a result of the various flow patterns in horizontal oil–water flow, the measurement response of capacitance sensors to the liquid holdup could be complicated and depends very much on flow structures. In addition, due to the leakage current effect caused by conductive water, the measurement sensitivity of capacitance sensors to the variation of the liquid holdup is low when water is encountered as a continuous phase in oil–water two-phase flow. Therefore, it is still a great challenge to design a high-sensitivity capacitive sensor system for water holdup measurement that has a low dependence on the complex horizontal oil–water flow structures.

Capacitance sensors have been widely used in the measurement of two-phase flows [1,2]. Ahmed et al. [3] and Salehi et al. [4] compared the performance of a ring-type capacitance sensor in void fraction measurement of oil-gas two-phase flow with that of a concave-type one. It was found that both sensors showed a linear relationship between their responses and the average void fraction, but the sensitivity of the ring-type sensor is higher than that of the concave type for the same spatial resolution. Ghai et al. [5] optimized and designed a helix capacitance sensor which possessed good water holdup measurement responses for both gas–liquid and oil–water stratified flows. Salehi et al. [6] designed a capacitance sensor with a new structure, i.e., twin rectangular fork-like capacitance (TRFLC), and compared the water holdup measurement characteristic of the TRFLC sensor with other typical capacitance sensors. It was found that the TRFLC sensor was more suitable for water holdup measurement in stratified flow. In practical applications, some scholars have applied capacitance sensors to the oil industry. Liu et al. [7] and Zhang et al. [8] used a coaxial capacitance sensor to measure the respective liquid holdup in vertical and horizontal oil–water two-phase flow. It was found that the sensor lost its sensitivity with high water holdup. Zhao et al. [9] and Zhai et al. [10] optimized the concave and helix capacitance sensors, respectively, by using the finite element method (FEM) to increase the sensor sensitivity and the accuracy for measuring the liquid holdup in horizontal oil–water two-phase flow. However, the optimized sensors still showed low sensitivity to high water holdup. Wu et al. [11] measured the water holdup of horizontal oil–water two-phase flow by using a capacitance-conductance combination method to compensate for the lack of capacitance sensors in high water holdup measurement. In addition, Demoria et al. [12] and Strazza et al. [13] investigated the influence of conductive water on the response characteristics of a concave capacitance sensor in the measurement of oil–water annular flow, and proposed solutions to parasitic coupling caused by stray elements outside the measurement section of the pipe. 

In recent years, new sensing technologies have been developed for two-phase flow measurement. For example, capacitively coupled contactless conductivity detection has been developed and used in the measurement of phase holdup in two-phase flow [14,15,16]. Notably, a method of phase shift detection [17] for two-phase flow based on a concave capacitance sensor (CCS) was designed by Jaworek et al. [18], and it was used to measure the void fraction of gas–liquid two-phase flow. A preferable linear relationship between the phase shift of the CCS and the void fraction was obtained, indicating that this method of phase shift detection has potential advantages for the measurement of nonuniform two-phase flow parameters. It should be noted that the liquid phase in the experiment of Jaworek et al. [18] is nonconducting distilled water. However, the structures of horizontal oil–water two-phase flow are complex and changeable, and the nonconducting oil phase and the conductive water phase can simultaneously serve as continuous phase, which results in great difficulties in the measurement of the liquid holdup. Using phase shift detection technology based on capacitance sensors to measure the liquid holdup of horizontal oil–water flows is a beneficial attempt. 

In this study, a phase shift detection system of flow impedance is designed based on a CCS to measure the water holdup of horizontal oil–water flows. Firstly, impedance structures of oil–water stratified flow are investigated by a comparative analysis of equivalent circuit analysis and the finite element method. The normalized results of the equivalent circuit analysis and the finite element method show a good consistency. Then, the parameters of the CCS are optimized by studying the influence of exciting frequency and electrode sizes on the CCS phase shift output. An experiment of horizontal oil–water two-phase flow was conducted. The experimental results indicate that the phase shift responses of the CCS present satisfied sensitivities for ST and SW flow patterns, and the phase shift response of the CCS shows better linearity and sensitivity for DC and DO/W&W as well. In general, the phase shift detection technology has advantages in improving the performance of CCS in water holdup measurement of two-phase flows with nonuniform phase distribution and conductive water-continued structures.

## 2. Model of Concave Capacitance Sensor

### 2.1. Structure of Concave Capacitance Sensor

**Figure 1:**
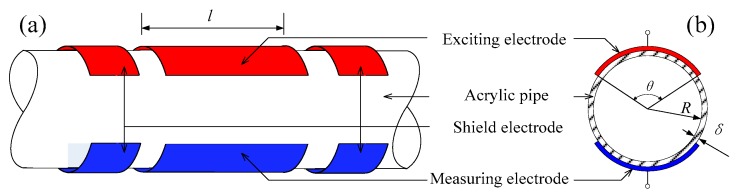
Structure of the concave capacitance sensor for capacitive phase shift detection. (**a**) 3D view; (**b**) cross section view.

The concave capacitance sensor (CCS) for the phase shift detection consists of an exciting electrode, a measuring electrode, and shield electrodes, as shown in Figure 1. All the electrodes are flush mounted on the outside of the pipe wall. The main geometric parameters of the CCS are the electrode length *l*, the electrode angle *θ*, the pipe wall thickness *δ*, and the pipe inner radius *R*.

### 2.2. Equivalent Circuit Model of CCS for ST Flow

**Figure 2:**
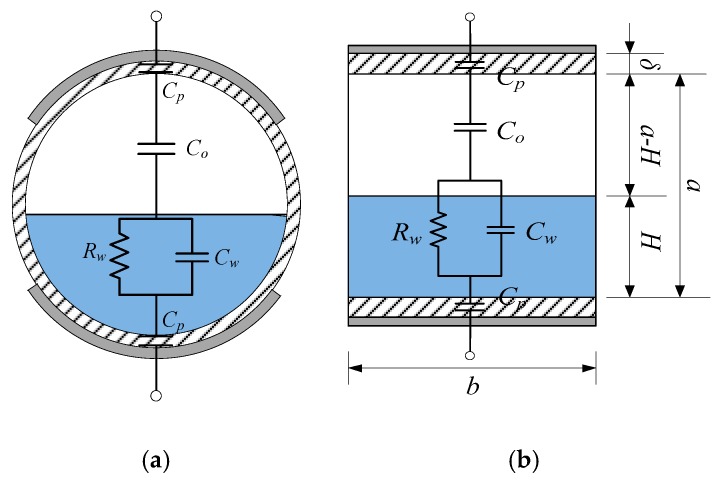
Impedance structure analysis of the concave capacitance sensor (CCS) for the stratified flow (ST) flow pattern: (**a**) equivalent impedance circuit model; (**b**) simplified impedance circuit model.

A 2D equivalent impedance circuit model of the CCS for the ST flow pattern is shown in Figure 2a. The equivalent impedance can be expressed as

(1) $$Z=\frac{2}{j\omega C_{p}}+\frac{1}{j\omega C_{o}}+\frac{1}{\frac{1}{R_{w}}+j\omega C_{w}}$$

where *Z* is the fluid impedance; *C_w_*, *C_o_*, and *C_p_* are the equivalent capacitance of water phase, oil phase, and the pipe wall; *R_w_* is the equivalent resistance of water phase; and *ω* is the angular frequency of the exciting signal. Equation (1) is rearranged as

(2) $$Z=\frac{R_{w}}{\omega^{2}C_{w}^{2}R_{w}^{2}+1}-j\left(\frac{2C_{o}+C_{p}}{\omega C_{p}C_{o}}+\frac{\omega C_{w}R_{w}^{2}}{\omega^{2}C_{w}^{2}R_{w}^{2}+1}\right).$$

It is obvious that the real part of the equivalent impedance is Re(Z)=Rwω2Cw2Rw2+1, whilst the imaginary part is Im(Z)=2Co+CpωCpCo+ωCwRw2ω2Cw2Rw2+1.

A simplified impedance circuit model for the ST flow pattern, as shown in Figure 2b, is built to simplify the calculation of the equivalent impedance *Z*. In this model, the pipe cross section is approximately transformed to a rectangle from a circle, where *a* represents the height of the rectangle; *b* is the width decided by the electrode size; *R* is the inner radius of the pipe cross section; *δ* is the pipe wall thickness; *l* is the electrode length; *θ* is the electrode angle; and *H* is equivalent water layer height. *a* and *b* are formulated according to the sensor geometric parameters:

*a* = 2*R* (3)

*b* = *θR*. (4)

The water holdup *H_w_* can be expressed using the water layer height *H* [19]:

(5) $$H_{w}=\{\begin{array}{ll} \frac{\arccos\left(\frac{R-H}{R}\right)}{\pi }+\frac{\left(R-H\right)\sqrt{2RH-H^{2}}}{\pi R^{2}} & H<R\\ 0.5 & H=R\\ \frac{\arccos\left(\frac{H-R}{R}\right)}{\pi }+\frac{\left(H-R\right)\sqrt{2RH-H^{2}}}{\pi R^{2}} & H>R \end{array}.$$

Thus, the electrical parameters of the equivalent impedance *Z* are expressed by

(6) $$C_{p}=\varepsilon_{0}\varepsilon_{p}\frac{bl}{\delta }$$

(7) $$C_{w}=\varepsilon_{0}\varepsilon_{w}\frac{bl}{H}$$

(8) $$C_{o}=\varepsilon_{0}\varepsilon_{o}\frac{bl}{a-H}$$

(9) $$R_{w}=\frac{1}{\sigma_{w}}\frac{H}{bl}$$

where *ε*_0_ = 8.854 × 10^−12^ F/m is the dielectric constant of vacuum; *ε_w_*, *ε_o_*, and *ε_p_* denote the relative dielectric constants of water phase, oil phase, and pipe wall which are selected as 80, 2.5, and 3.45, respectively; *σ_w_* denotes the conductivity of water phase and is selected as 0.03 S/m.

If we substitute Equations (6)–(9) into Equation (2), the equivalent impedance expression based on the simplified model can be obtained as

(10) $$Z=\frac{\sigma_{w}}{\omega^{2}\varepsilon_{0}^{2}\varepsilon_{w}^{2}+\sigma_{w}^{2}}\cdot \frac{H}{bl}-j\left[\frac{\varepsilon_{p}\left(a-H\right)+2\varepsilon_{o}\delta }{2bl\omega \varepsilon_{0}\varepsilon_{p}\varepsilon_{o}}+\frac{\omega \varepsilon_{0}\varepsilon_{w}}{\omega^{2}\varepsilon_{0}^{2}\varepsilon_{w}^{2}+\sigma_{w}^{2}}\cdot \frac{H}{bl}\right].$$

In Equation (10), the real part is Re(Z)=σwω2ε02εw2+σw2⋅Hbl, whilst the imaginary part is composed of the following three terms:

(11) Im1(Z)=−εoωε0εp⋅δbl∝δbl

(12) Im2(Z)=−ωε0εwω2ε02εw2+σw2⋅Hbl∝Hbl

(13) Im3(Z)=−12ωε0εo⋅a−Hbl∝a−Hbl.

These three terms of Im(*Z*) correspond to the capacitance of the pipe wall, the water phase, and the oil phase, respectively, as given by Equations (6)–(8).

### 2.3. Finite Element Model of CCS for ST Flow

**Figure 3:**
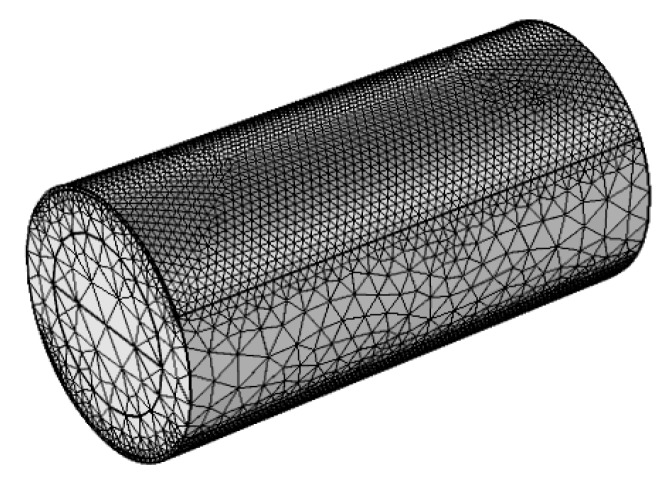
Meshed models of the CCS with water holdup of 50%.

The Finite Element Method (FEM) is widely used to simulate complex electric fields. In order to verify the validity of the equivalent circuit model, a 3D finite element model of the CCS was constructed by using COMSOL Multiphysics software, as seen in Figure 3. A frequency domain module is used to simulate the impedance of the CCS under an AC excitation. Maxwell’s equations are solved to determine the electric field distribution as follows [20]:

(14) $$\nabla \times \left(\frac{1}{\mu^{\prime }}\nabla \times \vec{E}\right)-\frac{\omega^{2}}{c}\left(\varepsilon^{\prime }-i\varepsilon^{\prime \prime }\right)\vec{E}=0$$

where $\vec{E}$ is electric field intensity, $\varepsilon^{\prime }$ is relative dielectric constant of the medium, $\varepsilon^{\prime \prime }$ is relative dielectric loss of the medium, *ω* is angular frequency, $\mu^{\prime }$ is relative permeability of the medium, and *c* is speed of light in vacuum.

**Figure 4:**
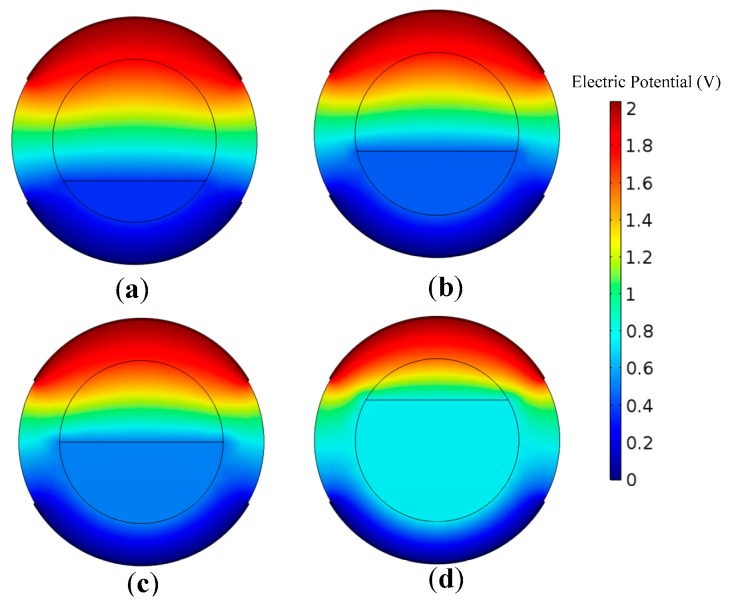
Cross section potential distribution of the CCS calculated by COMSOL Multiphysics. (**a**) *H_w_* = 20%; (**b**) *H_w_* = 30%; (**c**) *H_w_* = 50%; (**d**) *H_w_* = 80%.

The finite element model consists of an acrylic pipe and two copper electrodes. The inner diameter of the pipe was set as 20 mm, and the pipe wall thickness was set as 5 mm. The thickness of the electrodes was set as 0.5 mm, and the electrode angle was set as 120°. The flow channel is divided into upper and lower parts, and the two parts are set as oil and water phase, respectively. The location of the divisional plane is decided by different water heights corresponding to the water holdup (see Equation (5)). The top electrode serves as the exciting electrode, which is supplied by an exciting voltage with amplitude of 2.00 V and frequency of 1 MHz. Meanwhile, the bottom electrode is grounded. Figure 4 shows the calculated potential distributions of the CCS at the pipe cross section, with different water holdups. As can be seen, the potential close to the exciting electrode is the highest, and decreases with increasing distance from the exciting electrode. However, the potential seldom changes in the water phase which forms an equipotential body.

**Figure 5:**
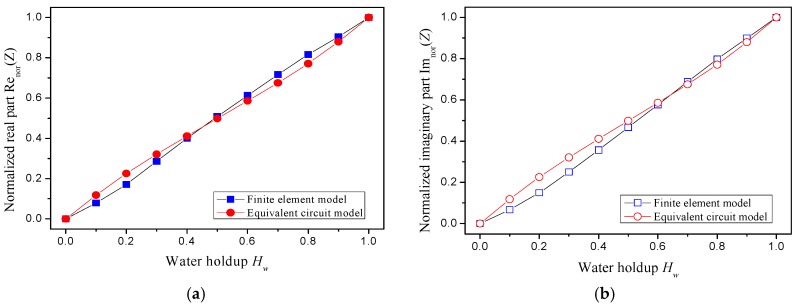
The normalized impedance from the finite element model and equivalent circuit model: (**a**) real part; (**b**) imaginary part.

The impedances of stratified oil–water flows are calculated by using the equivalent circuit model and the finite element model. Figure 5 shows the normalized results of the two models by using Equations (15) and (16):

(15) Renor(Z)=Re(Z)−Re(Zo)Re(Zw)−Re(Zo)

(16) Imnor(Z)=Im(Z)−Im(Zo)Im(Zw)−Im(Zo)

where Re_nor_ and Im_nor_ respectively denote the normalized real part and imaginary part of the flow impedance, and *Z_o_* and *Z_w_* respectively denote the flow impedance when the pipe is filled with oil and water. As can be seen, the normalized real and imaginary parts of the flow impedance both increase with the water holdup increasing, and present a good linearity. Notably, the normalized flow impedances from both models have good consistency. We can conclude that the equivalent circuit model has potential for uncovering the flow impedance structures, and, thus, the CCS geometry is optimized using equivalent circuit analysis in the section below.

## 3. Design of the Phase Shift Detection System

### 3.1. Phase Shift Detection System

**Figure 6:**
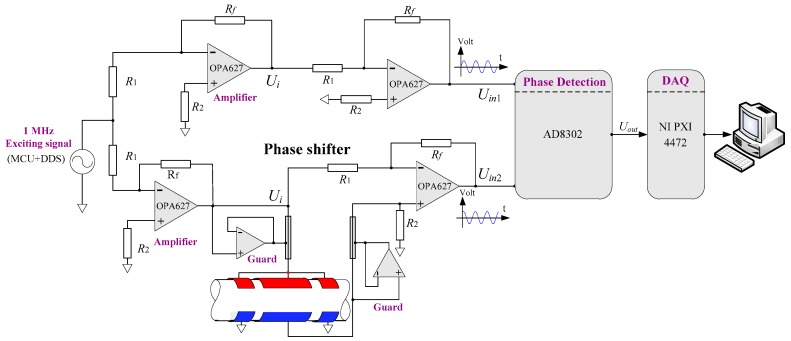
Schematic diagram of the measurement system for capacitive phase shift detection.

The schematic diagram of the measurement system for capacitive phase shift detection is shown in Figure 6. This system consists of an exciting signal circuit, a phase shift circuit, a reference amplifying circuit, a phase shift detection circuit, and a data acquisition device. The exciting signal is selected as a sine voltage with a frequency of 1 MHz and is generated by a direct digital synthesizer (AD9831, ADI Company, Norwood, MA, USA) which is controlled by a microcontroller unit (ATmega16, Atmel Company, San Jose, CA, USA). After a process of reversing amplification, the exciting signal is simultaneously input to the phase shift circuit and the reference amplifying circuit. The upper shield electrodes are connected with the exciting voltage, and the bottom ones are grounded to overcome the edge effect of the electric field and to reduce the leakage current. When oil and water flow through the CCS, the capacitance of the CCS will produce a corresponding change and, thus, there will be a phase shift in the output of the phase shift circuit. This phase shift is detected by the phase shift detection circuit which is designed using an AD8302 IC chip (ADI Company). The AD8302 enables us to provide an accurate measurement of the phase shift over 0° to 180°. Specifically, the output voltage of AD8302 shows a linear increase from 0.9 V to 1.8 V within the phase shift angle of −90° to 0° and a linear decrease from 1.8 V to 0.9 V within the phase shift angle of 0° to 90°. The output of the phase shift detection circuit is collected by a data acquisition (DAQ) device (PXI4472, NI Company, Austin, TX, USA). The LabView 7.1 software on the host computer provides functions of real-time data waveform display, storage, and analysis. 

### 3.2. Basic Phase Shift Circuit

**Figure 7:**
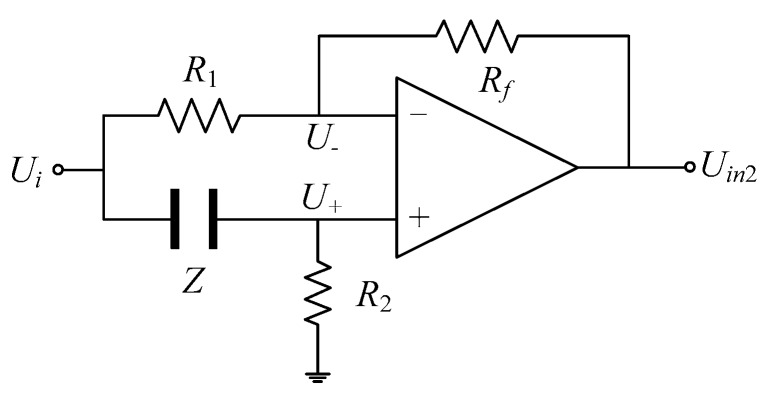
Schematic diagram of basic phase shift circuit.

The schematic diagram of the basic phase shift circuit is shown in Figure 7, in which *U_i_* and *U_in_*_2_ represent the input and the output voltage of the phase shift circuit, respectively; *R*_1_, *R*_2_, and *R_f_* are fixed resistances; and *Z* is the equivalent impedance of the CCS. According to the virtual short characteristic of the operational amplifier, it is obvious that

(17) $${\dot{U}}_{+}={\dot{U}}_{-}.$$

Thus,

(18) $$\frac{R_{2}}{Z+R_{2}}{\dot{U}}_{i}=\frac{R_{f}}{R_{1}+R_{f}}\left({\dot{U}}_{i}-{\dot{U}}_{in2}\right)+{\dot{U}}_{in2}.$$

If $k=\frac{R_{f}}{R_{1}+R_{f}}$, Equation (18) can be written as

(19) $$\frac{R_{2}}{Z+R_{2}}{\dot{U}}_{i}=k\left({\dot{U}}_{i}-{\dot{U}}_{in2}\right)+{\dot{U}}_{in2}.$$

Equation (19) can be rearranged as

(20) $$\frac{{\dot{U}}_{in2}}{{\dot{U}}_{i}}=\frac{\left(k-1\right)R_{2}+kZ}{\left(k-1\right)\left(R_{2}+Z\right)}.$$

The equivalent impedance Z=Re(Z)+jIm(Z) is substituted into Equation (20):

(21) U˙in2U˙i=(k−1)R2+k[Re(Z)+jIm(Z)](k−1)[R2+Re(Z)+jIm(Z)]=[(k−1)R2+kRe(Z)][R2+Re(Z)]+kIm2(Z)+jR2Im(Z)(k−1){[R2+Re(Z)]2+Im2(Z)}.

Thus, the tangent value of the phase shift angle Δ*ϕ* between the output voltage and input voltage can be described as

(22) tan(Δφ)=Im(U˙in2U˙i)Re(U˙in2U˙i)=R2Im(Z)[(k−1)R2+kRe(Z)][R2+Re(Z)]−kIm2(Z).

In the phase detection circuit, the input voltages of the AD8302 are *U_in_*_1_ and *U_in_*_2_ with phase difference $\Delta \varphi^{\prime }$. If the phases of *U_in_*_1_, *U_in_*_2_, and *U_i_* are respectively denoted by *ϕ_in_*_1_, *ϕ_in_*_2_, and *ϕ_i_*, we can obtain Equation (23) according to the circuit in the Figure 6.

(23) $$\{\begin{array}{c} \Delta \varphi =\varphi_{in2}-\varphi_{i}\\ \Delta \varphi^{\prime }=\varphi_{in2}-\varphi_{in1}\\ \pi =\varphi_{in1}-\varphi_{i} \end{array}$$

We thus obtain

(24) $$\Delta \varphi -\Delta \varphi^{\prime }=\pi .$$

Since the absolute value of the input phase difference and output voltage of AD8302 present a periodic relationship with period *π* (see the specification of AD8302), the output of the phase shift detection circuit enables us to reflect the phase shift Δ*ϕ* caused by the fluid in CCS.

## 4. Parameter Optimization of the Sensor 

**Figure 8:**
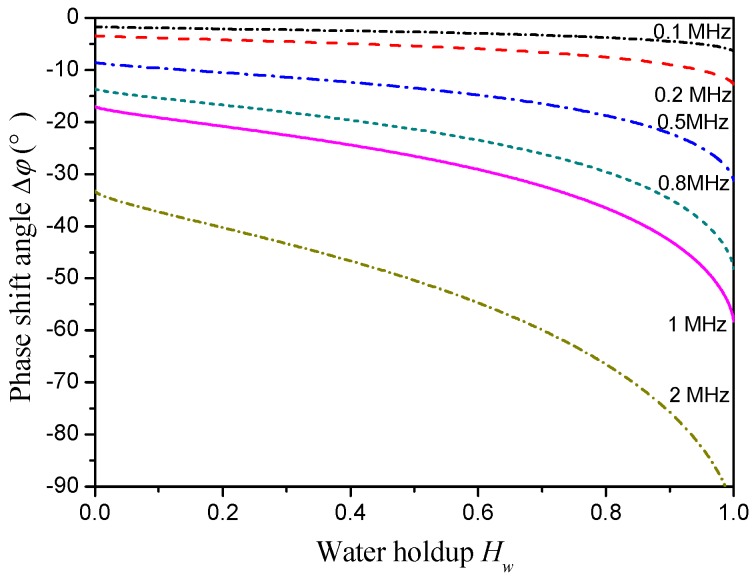
Phase shift responses of the CCS with different exciting frequencies.

The effects of exciting signal frequency and sensor geometrical sizes on the phase shift response characteristics of the CCS are investigated under ST flow by substituting the equivalent circuit model into the basic phase shift circuit. The phase shift responses of the CCS with different exciting signal frequencies are shown in Figure 8. The phase shift angles are negative for all the investigated exciting signal frequencies and decrease with increasing water holdup. Meanwhile, the decreasing rate of the phase shift angle depends on the exciting frequencies. Specifically, a higher exciting frequency could lead to a high sensitivity of the phase shift angle to the variation of the water holdup. For the exciting frequency of 2 MHz, the phase shift angle is lower than −90° at high water holdup. This low phase shift angle will result in a nonmonotonic output from the AD8302. Thus, in this study, the exciting signal frequency is selected as 1 MHz.

**Figure 9:**
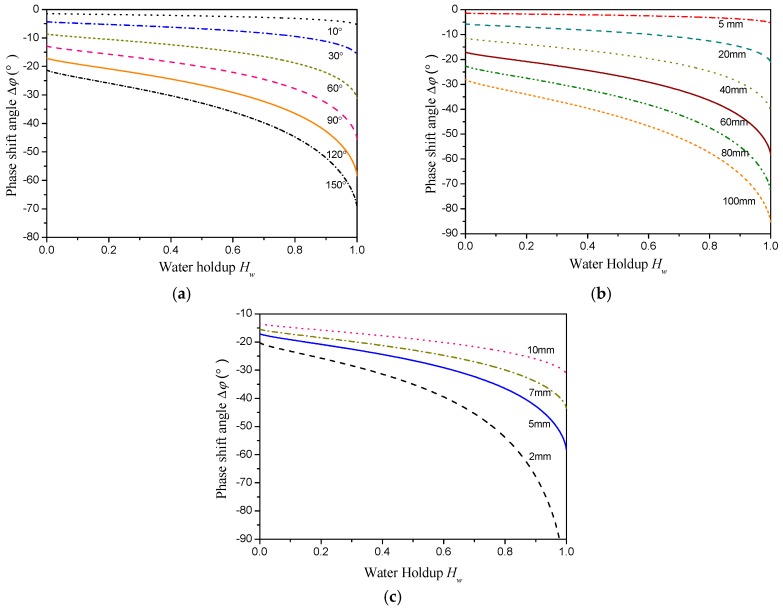
Phase shift responses of the CCS with different geometric parameters. (**a**) With different electrode angles; (**b**) with different electrode lengths; (**c**) with different pipe wall thicknesses.

Figure 9a–c show the phase shift responses of the CCS with different electrode angles, electrode lengths, and pipe wall thicknesses for the ST flow pattern. The variation range of the phase shift indicates an increasing tendency with the increase in the electrode angle *θ* and the electrode length *l*, but a decreasing tendency with the increase in the pipe wall thickness *δ*. It should be noted that for a circular pipe cross section, a larger electrode angle can produce an obvious edge effect, and a longer electrode length can lead to low detection sensitivity for small-scale flow structures. Therefore, in this study, the electrode angle *θ* is selected as 120° and the electrode length *l* is selected as 60 mm. Since the pipe should be strong enough to avoid damage in the flow loop test, the pipe thickness *δ* is selected as 5 mm. The shield electrodes use the same geometrical parameters as the exciting and measuring electrodes.

**Figure 10:**
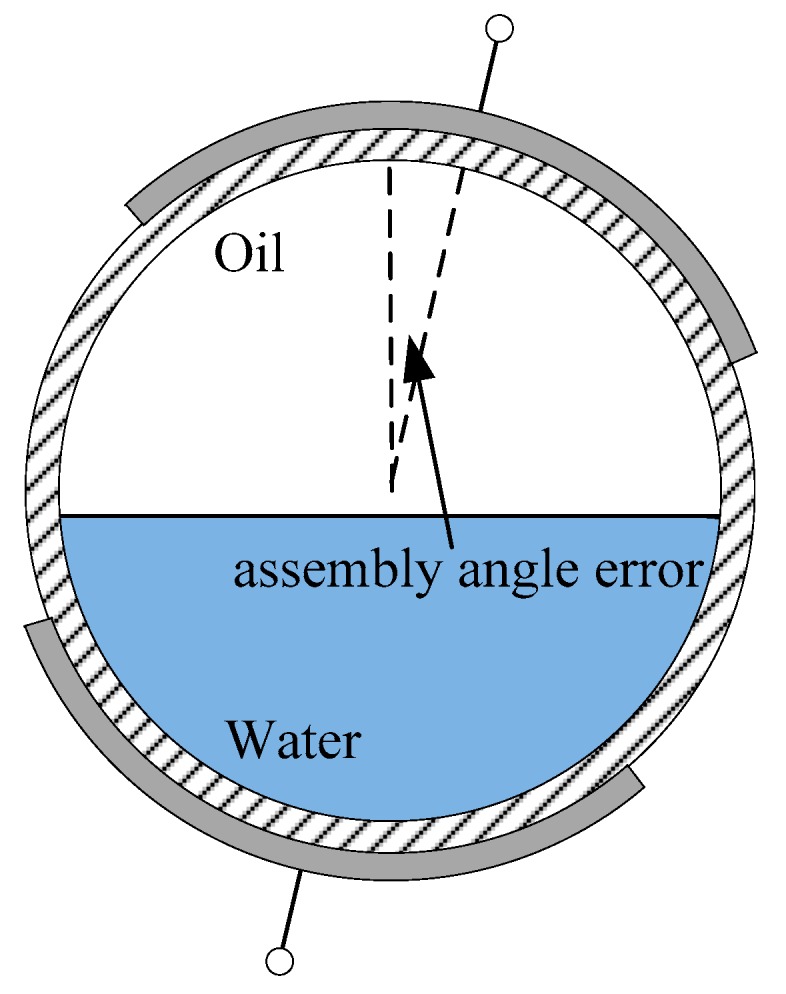
Sketch of assembly angle error during sensor installation.

The electrodes of the CCS should be set at the top and bottom of the pipe. However, there may be an assembly angle error from the vertical during sensor installation, as shown in Figure 10. The assembly angle error is also a considerable factor affecting sensor phase shift output. For evaluating the effect of assembly angle error on the sensor phase shift outputs, a finite element calculation of the sensor response with the assembly angle changing within a certain range is carried out. The calculated results are presented in Table 1. It can be seen that, compared with the result without assembly angle error, the error of the calculated phase shift output is less than 0.4% when the assembly error angle is less than 5°. This result demonstrates that the phase shift output of the CCS is credible for a slight assembly angle error.

## 5. Experiment with Oil–Water Two-Phase Flow

### 5.1. Experimental Setup

**Figure 11:**
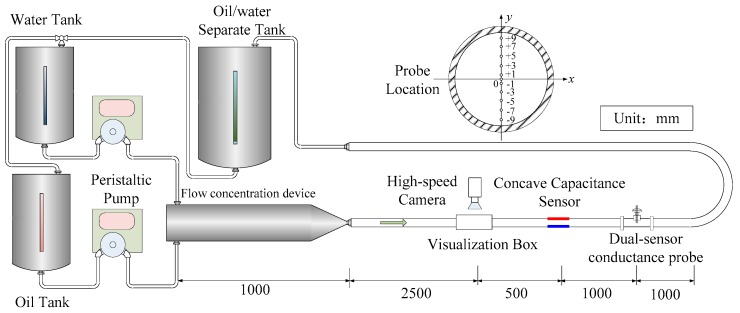
Schematic diagram of the experimental setup for horizontal oil–water two-phase flow.

An experiment with horizontal oil–water two-phase flow was carried out in the multiphase flow laboratory of Tianjin University. The experimental setup is schematically shown in Figure 11. The experimental media were tap water and 3# industry white oil. The densities of the water and oil phases are, respectively, 1000 and 801 kg/m^3^, while the viscosities of the water and oil phases are, respectively, 1 and 3.5 mPa·s. The test section was made up of an acrylic pipe and its inner diameter was 20 mm. Oil and water were forced into the test section through a flow concentration device. In the experiment, the oil flow superficial velocity ranged over 0.022, 0.067, 0.11, 0.195, 0.3, 0.432, and 0.51 m/s and the water flow superficial velocity ranged over 0.052, 0.11, 0.166, 0.222, 0.28, 0.336, 0.393, 0.45, 0.51, 0.563, and 0.62 m/s. For each flow condition, the oil flow rate was fixed and then the water flow rate was gradually increased from 0.052 to 0.62 m/s. A high-speed camera, a CCS, and a dual-sensor conductance probe were successively mounted in the test section. According to the study by Zhai et al. [19], oil–water two-phase flow can fully develop at the location of 1200 mm away from the inlet, due to the existence of the flow concentration device. Hence, the visualization box of the high-speed camera was located at 2500 mm behind the flow concentration device. The flow structures can be directly observed on the images captured by the camera. The CCS was fixed at 500 mm after the camera. The dual-sensor conductance probe was mounted at 1000 mm behind the CCS to measure the local parameters of oil–water two-phase flow.

The dual-sensor conductance probe is sensitive to local conductivity characteristics of oil–water two-phase flow. The dual-sensor conductance probe is composed of two conductive probes and a position adjustment device. The measurement signal is low in level when the tips of the probe are immersed in oil phase. By contrast, the probe produces a high level when its tips are in water phase. For the measurement principle of the dual-sensor conductance probe, the reader can refer to Zhai et al. [21,22]. A transverse mechanism is designed to control the position of the probe tips along the vertical direction of the pipe. As shown in Figure 11, the measurement of the dual-sensor probe was taken from the bottom of the pipe for every 2 mm towards the top. The high-speed camera in the experiment is based on complementary metal-oxide semiconductor (CMOS) sensing technology, and its pixel size is 7 μm × 7 μm. The camera resolution was set as 2320 × 600. The oil phase was dyed red to distinguish oil from water clearly in the images. In black-and-white images obtained by the camera, the dark part represents the oil phase, and the light part represents the water phase.

### 5.2. Experimental Results and Discussions

The flow patterns encountered in our experiment include stratified flow (ST), stratified wavy flow (SW), dual continuous flow (DC), and dispersion of oil-in-water and water flow (DO/W&W). In ST flow, the oil and water phases flow in the form of two layers with the oil layer flowing above the water layer, and the oil–water interface is smooth and stable. In SW flow, both phases are still segregated, but at the interface appear waves with considerable amplitudes. When the flow develops to DC flow, the oil phase and the water phase are both continuous and entrain a large number of water and oil droplets, respectively. In the DO/W&W flow, the oil phase distributes in the upper part of the pipeline in the form of dispersed droplets, and the water is the continuous phase. For detailed descriptions of flow patterns, the reader can refer to Zhai et al. [19] and Edomwonyi-Otu et al. [23].

**Figure 12:**
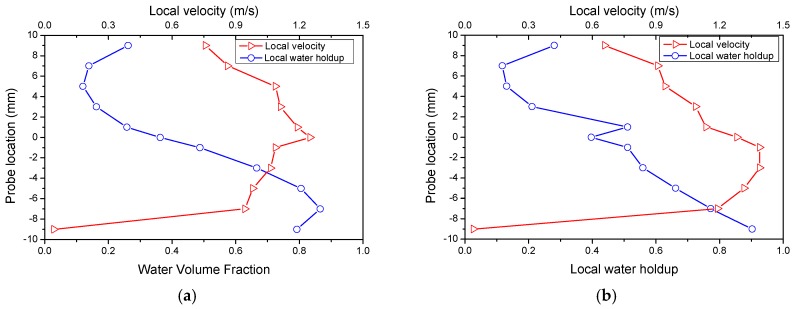
The profile of local velocity and local concentration in representative conditions with fixed oil phase superficial velocity *U_so_* = 0.51 m/s. (**a**) *U_sw_* = 0.51 m/s, dual continuous (DC) flow; (**b**) *U_sw_* = 0.62 m/s, DC flow.

The horizontal oil–water two-phase flow is characterized by different profiles of local velocity and local water holdup. The local velocity and the local water holdup were obtained by the dual-sensor conductance probe. At each probe location, oil phase and water phase alternate through the probe tip. The ratio of the water phase duration to the acquisition time can be defined as the local water holdup at the probe location [21]. The local velocity is calculated by the distance and the transit time calculated by the cross-correlation function [22]. Figure 12 shows the measurement profiles of the local velocity and local water holdup of DC flows. As seen, the water holdup profiles have a similar “S” shape. The water holdup increases gradually from top to bottom, except for positions near the pipe wall. The local velocities in DC flow have obvious nonuniform distributions. Specifically, the local velocity at the pipe center is much higher than that around the pipe wall, and the maximum velocity is always encountered at the oil–water interface. Notably, in DC flow, both phases can simultaneously serve as the continuous phase. The complex and changeable structures of the oil–water flow result in great challenges in the measurement of the liquid holdup.

**Figure 13:**
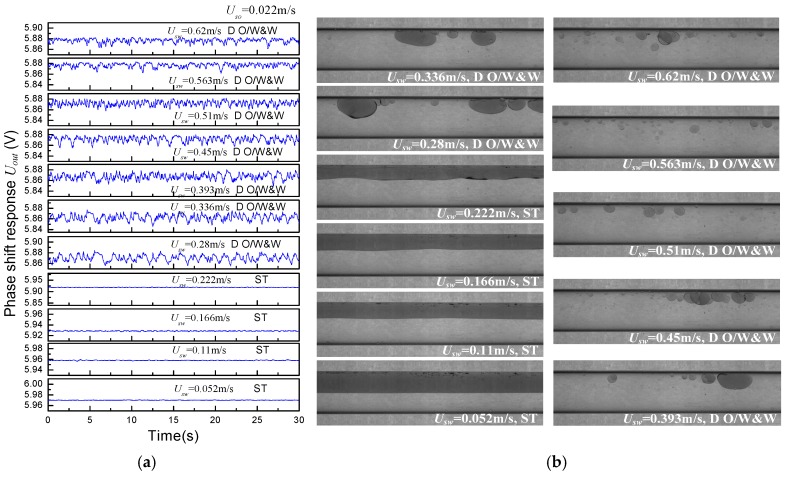
Phase shift response of (**a**) the CCS and (**b**) oil–water images (*U_so_* = 0.022 m/s).

**Figure 14:**
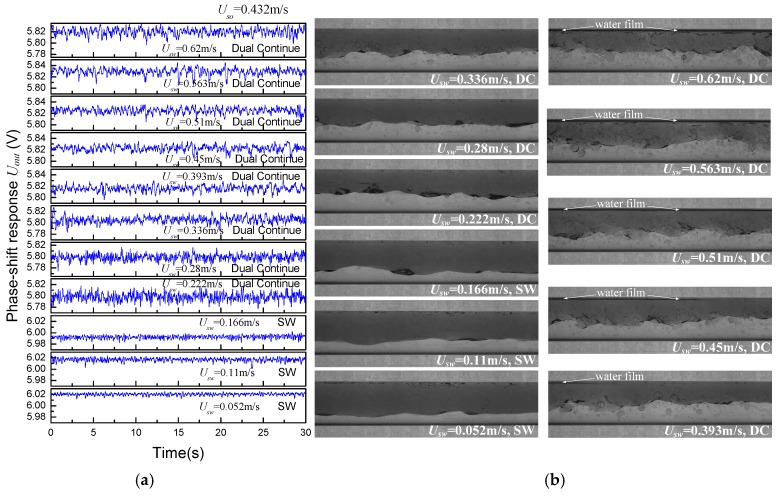
Phase shift response of (**a**) the CCS and (**b**) oil–water images (*U_so_* = 0.432 m/s).

Figure 13 and Figure 14 present the phase shift responses of the CCS under typical flow conditions. Generally, the signal fluctuation grows with increasing water superficial velocity, when the oil superficial velocity *U_so_* is constant. As shown in Figure 13a, no obvious changes in the sensor responses are observed when *U_sw_* is lower than 0.222 m/s. As the water superficial velocity increases, the flow pattern transforms to DO/W&W flow from ST flow. Accordingly, the fluctuation range of the sensor responses is extended significantly, but the fluctuation frequency remains low. As seen in the oil–water images, when *U_sw_* is equal to 0.28 m/s, the oil phase appears in the form of intermittent oil slug. As *U_sw_* increases to 0.45 m/s and 0.62 m/s, the oil phase is dispersed into smaller droplets with higher motion frequency. Thus, the fluctuation frequency of the CCS responses presents an obvious increase, whilst the fluctuation amplitude decreases accordingly. 

For the flow conditions of *U_so_* = 0.432 m/s, as shown in Figure 14, a low water superficial velocity can generate large-amplitude interfacial waves which result in large signal fluctuations. With increasing water flow rate, fully developed DC flow occurs. We can see large-scale interface waves and complicated droplet entrainment in DC flows. Accordingly, the CCS responses show high-frequency and large-range fluctuations. It should be stressed that a thin water film can be observed near the upper pipe wall, leading to a different water wetting behavior compared with SW and ST flow.

**Figure 15:**
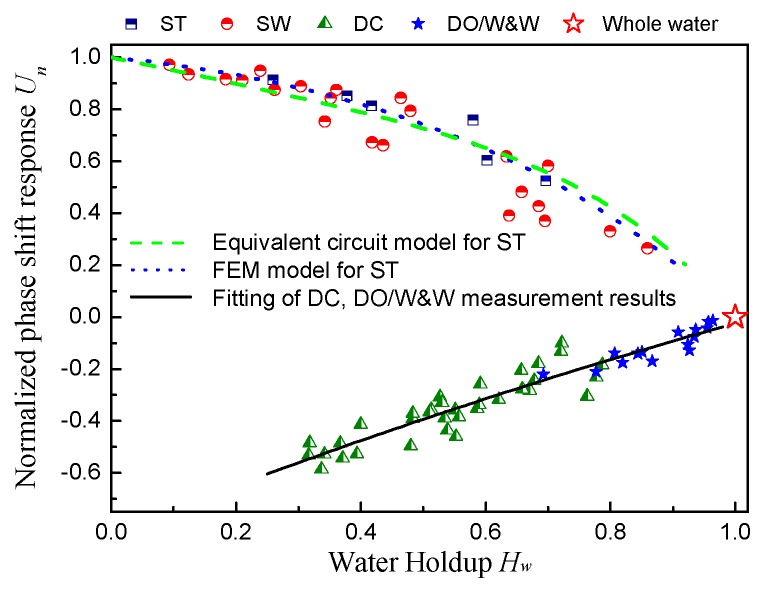
Phase shift response characteristics of the CCS in horizontal oil–water two-phase flow. The red star symbol denotes the sensor response when the pipe is filled with water.

The phase shift response characteristics of the CCS are normalized using Equation (25) and presented in Figure 15:

(25) $$U_{n}=\frac{U_{out}-U_{water}}{U_{oil}-U_{water}}$$

where *U_n_* is the normalized phase shift response, *U_out_* is the CCS average output voltage collected by the DAQ in the acquisition time of 30 s, and *U_oil_* and *U_water_* represent the output voltage when the pipe is filled with oil or water, respectively. The water holdup is the mean value of the local water holdup at the 11 probe locations in the condition.

For ST and SW flow, we can see that there is a favorable monotone relation between the normalized phase shift response and the water holdup, and this response shows a decreasing tendency when the water holdup increases. Figure 15 exhibits the normalized results from the equivalent circuit model and the FEM model for the ST flow as well. As can be seen, the calculated results of the two models are in line with the measurement results for ST flow. For SW flow, the measurement results are in accord with the model results for ST flow when the water holdup is lower than 40%. However, there is a deviation between the model results and the measurement results for SW flow with high water holdup. This deviation results from the fact that the interfacial wave is neglected in the modeling of the flow impedance. 

The normalized phase shift response of the CCS for DC and DO/W&W flow shows an increasing tendency with water holdup increasing. According to the visualizations of DC flow, an obvious water film wetting the inner pipe wall can be frequently observed. This special pipe wetting phenomenon leads to the result that the impedance structure of DC flow is similar to that of DO/W&W flow. In this case, the presence of the continuous water phase produces conductive paths that couple the measurement section between the electrodes with the outer portions of the pipe [12]. Hence, there will be a leakage current phenomenon in DC and DO/W&W due to the conductive water. The leakage current can lead to an increase of the equivalent impedance between the electrodes. The variation of the equivalent impedance can make the phase shift angle less than −90°, and, thus, the phase shift angle enters into the interval of [90°, 0°] with respect to the phase shift circuit period of *π*. According to the output characteristic of AD8302 (see the specification of AD8302), the output signal of the measurement system therefore presents an increasing tendency with the phase shift angle decreasing, which is caused by the increasing water holdup.

Accordingly, it can be seen from Figure 15 that the normalized phase shift responses to DC and DO/W&W flows increase with the rising water holdup. Notably, the phase shift response of the CCS exhibits satisfied linearity and sensitivity for DC flow and DO/W&W flow. This result indicates that the phase shift detection technology has advantages in improving the performance of CCS in water holdup measurement of two-phase flows with nonuniform phase distribution and conductive water-continued structures.

**Figure 16:**
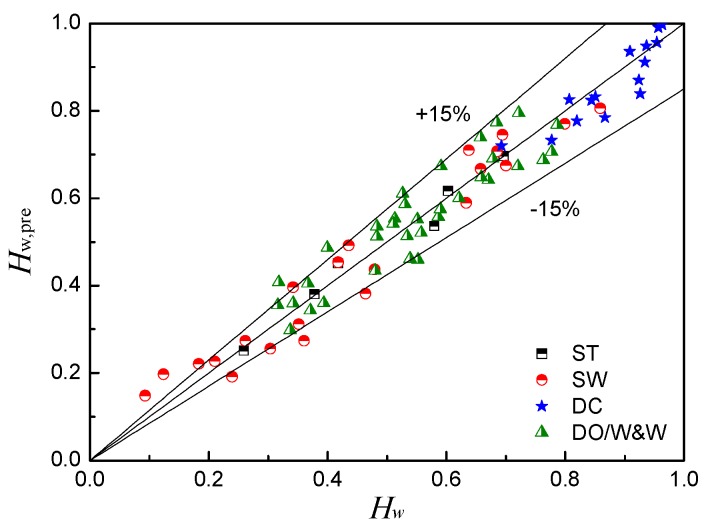
Prediction results of the water holdup using the phase shift detection technology.

The holdup prediction models of horizontal oil–water two-phase flow are obtained by fitting the normalized phase shift response of the CCS, as shown in Table 2. Figure 16 shows the prediction results of the water holdup using the phase shift detection technology. The average relative errors for the water holdup prediction are 3.576%, 15.058%, and 7.569% for ST, SW, and DC flow, respectively. This result indicates that the phase shift detection technology base on the CCS is a beneficial way to achieve the water holdup measurement of horizontal oil–water two-phase flow. Notably, even for DC and DO/W&W flows with complicated phase distribution and entrained multiscale droplets, the phase shift responses of the CCS exhibit good sensitivity and linearity with the variation of the water holdup, indicating the advantages of the capacitive phase shift technology in the measurement of nonuniform two-phase flow. In addition, we find that the phase shift responses of the CCS are dependent on the wave characteristics of the oil–water interface, which results in a lower accuracy of the water holdup prediction for SW flows.

## 6. Conclusions

In this paper, a phase shift detection system based on a concave capacitance sensor (CCS) is designed to measure the water holdup of horizontal oil–water two-phase flow. An equivalent circuit model and a finite element model are established for analyzing the flow impedance, and the two models present satisfactory consistency. The sensor geometry and the exciting signal frequency are optimized by using equivalent circuit analysis according to their influence on the phase shift output of the CCS. 

A horizontal oil–water two-phase flow experiment was carried out in the multiphase flow laboratory of Tianjin University. Four kinds of flow patterns were observed in the experiment, i.e., ST flow, SW flow, DC flow, and DO/W&W flow. The phase shift responses of the CCS for the different flow patterns were measured in the experiment. It was found that the phase shift response presents good linearity and sensitivity to the variation of the water holdup in ST and SW flow. For DC flow, the phase shift response characteristics of the CCS are similar to that for DO/W&W flow, which is probably caused by the water film appearing at the upper part of the inner pipe wall. 

It should be stressed that, even for DC and DO/W&W flows which are characterized by conductive and continued water phase and nonuniform distribution of the flow structure, the phase shift responses of the CCS present good performance in water holdup measurement, indicating the advantages of the capacitive phase shift technology in the measurement of two-phase flow with complicated flow structures. In addition, the phase shift responses of the CCS are dependent on the wave characteristics of the oil–water interface. Improving the measurement accuracy of the phase shift detection system for large-scale interfacial motion is the part of our work that has been identified for further investigation. 

## Figures and Tables

**Table 1:** The phase shift output (*ϕ*) of CCS with change in assembly angle error (using the optimized parameters).

*H_w_*	No Error	3° Assembly Angle Error		5° Assembly Angle Error		10° Assembly Angle Error	
	*ϕ* (°)	*ϕ* (°)	Relative Error	*ϕ* (°)	Relative Error	*ϕ* (°)	Relative Error
10%	−17.96	−17.97	0.05%	−17.98	0.13%	−18.05	0.52%
20%	−18.98	−19.00	0.10%	−19.03	0.27%	−19.19	1.08%
30%	−20.39	−20.41	0.12%	−20.46	0.32%	−20.65	1.27%
40%	−22.15	−22.17	0.13%	−22.22	0.34%	−22.44	1.33%
50%	−24.26	−24.29	0.13%	−24.35	0.36%	−24.35	0.36%
60%	−26.84	−26.88	0.14%	−26.94	0.39%	−27.25	1.55%
70%	−30.03	−30.07	0.13%	−30.14	0.38%	−30.47	1.45%
80%	−34.01	−34.04	0.08%	−34.12	0.32%	−34.37	1.05%
90%	−38.72	−38.73	0.02%	−38.74	0.04%	−38.79	0.18%

**Table 2:** Water holdup prediction model based on phase shift response.

Flow Pattern	Prediction Model
ST	$H_{w,\mathrm{pre}}=0.12447+0.23712U_{n}-3.12167U_{n}^{2}$
SW	$H_{w,\mathrm{pre}}=1.00477-0.2964U_{n}-0.61932U_{n}^{2}$
DC and DO/W&W	$H_{w,\mathrm{pre}}=1.02588+1.31161U_{n}-1.08193U_{n}^{2}$
